# Saikosaponin-d Increases the Radiosensitivity of Hepatoma Cells by Adjusting Cell Autophagy

**DOI:** 10.7150/jca.30286

**Published:** 2019-08-27

**Authors:** Yin-Di Tian, Shuai Lin, Peng-Tao Yang, Ming-Hua Bai, Ying-Ying Jin, Wei-Li Min, Hong-Bing Ma, Bao-Feng Wang

**Affiliations:** 1 Department of Infectious Diseases, the Second Affiliated Hospital of Medical College, Xi'an Jiaotong University, Xi'an 710004, China; 2 Department of Surgical Oncology, Second Affiliated Hospital of Medical School, Xi'an Jiaotong University, Xi'an 710004, China; 3 Department of Radiation Therapy, Second Affiliated Hospital of Medical School, Xi'an Jiaotong University, Xi'an 710004, China

**Keywords:** Saikosaponin-d, Radiation, Hepatocellular carcinoma, Autophagy.

## Abstract

Radiotherapy for liver cancer can affect the level of autophagy in cells, and effective autophagy regulation can increase the radiosensitivity of liver cancer cells.Saikosaponin-d (SSd) is an effective active ingredient extracted from traditional Chinese medicine Bupleurum. We have confirmed previously in vitro and in vitro experiments that SSd can significantly induce apoptosis of liver cancer cells, increase the radiosensitivity of liver cancer cells.This study explored the role of autophagy in SSd-mediated radiosensitivity of liver cancer cells. MTT and clone formation experiments showed that radiation can inhibit the proliferation of hepatoma cells and reduce the colony formation of hepatoma cells. After the addition of SSd, the inhibitory effect of radiation on the proliferation and clonal formation of hepatoma cells was further enhanced. However, the addition of the autophagy inhibitor chloroquine or mTOR agonist can partially reverse the inhibitory effect of the combined treatment of SSd with radiation on the proliferation of hepatoma cells. Similarly, transmission electron microscopy and laser confocal microscopy showed that after the addition of SSd, the number of radiation-induced autophagosomes increased significantly in hepatoma cells and the intervention of mTOR agonist can reduce the formation of autophagosomes in hepatoma cells.In addition,Western blot analysis presented that radiation significantly increased LC3-II levels. Especially when SSd is added, LC3-II levels is further increased. Our data indicate that SSd can inhibit the growth of liver cancer cells and enhance cell radiosensitivity by inducing autophagy formation.

## Introduction

Hepatocellular carcinoma is prevalent worldwide, and is one of the major diseases that severely threat the human health. The morbidity and mortality of liver cancer present an increasing trend 1,2. According to WHO statistics, the annually new patients of liver cancer reach about 1 million people all around the world. In China, the annually new patients of liver cancer also take up a proportion of 41% among all malignancies 3,4. Traditional treatments for liver cancer mainly include surgical resection, liver transplantation, radiofrequency ablation, embolization chemotherapy and so on. But all of these treatments are not effective, and the 5-year survival rate of liver cancer is only about 5%. In recent years, radiotherapy has proven to be a safe and effective treatment in liver cancer, especially stereotactic radiotherapy has made great progress in the treatment of liver cancer 5,6.

Radiotherapy of liver cancer can affect the level of autophagy in cells. Effective regulation of autophagy can increase the radiosensitivity of liver cancer cells 7,8. Therefore, further study of the role of autophagy is of great significance for the treatment of liver cancer. Autophagy is a protein degradation pathway used by eukaryotic cells to remove intracellular damage, and maintain homeostasis, which plays an important role in the physiology and pathology of cells, especially in the development of malignant tumors. However, excessively upregulated autophagy can also cause autophagic cell death, also known as type II programmed cell death, which is a programmed cell death pathway different from apoptosis 9,10.

Kuwahara et al. 11 found that the autophagy inducer, rapamycin, can enhance the radiosensitivity of radioresistant liver cancer cell HepG2 and inhibit the proliferation of tumor cells, while the autophagy inhibitor 3-MA enhances the radioresistance of cells. At the same time, the expression of the autophagy gene Beclin-1 was reduced. Similarly, expression of the exogenous autophagy gene Beclin-1 induces autophagy in tumor-resistant lung cancer cells and inhibits tumor cell growth and angiogenesis 12.

Saikosaponin-d (SSd) is an effective active ingredient extracted from traditional Chinese medicine Bupleurum. In the early stage, we confirmed in vitro and in vitro experiments that SSd can significantly induce apoptosis of liver cancer cells, increase the radiosensitivity of liver cancer cells, and inhibit the growth of liver tumors 13,14. Wong et al 15 found that SSd is an autophagy inducer that promotes autophagy by regulating the AMPK-mTOR pathway and induces autophagic death in liver cancer cells. However, the effect of SSd on the level of autophagy in liver cancer cell radiotherapy has not been reported.

Therefore, based on our previous experimental results, this study was to observe the inhibitory effect of SSd and radiation alone and in combination on the growth of hepatoma cells, and to detect the changes of autophagy and related proteins. This study preliminarily explored the role of autophagy in SSd-mediated radiosensitivity of hepatoma cells, and provided experimental evidence for the treatment of liver cancer and the development of new drugs.

## Materials and Methods

### Drug and reagents

Chloroquine (CQ), 3-(4,5-dimethyl-2-thiazolyl)-2,5- diphenyl-2-H-tetrazolium bromide (MTT), MHY1485 and dimethylsulfoxide (DMSO) were provided by Sigma Chemical Co. (St. Louis, MO,USA).SMMC-7721, MHCC97L human HCC cell lines, and normal liver cell line HL7702 (L02)were purchased from the Medical Experimental Animal Center of the Fourth Military Medical University (Xi'an, China); Saikosaponin-d was purchased from Bencao Tiangong Technology Co.,Ltd(Jiangxi, China). The GFP-RFP-LC3 fluorescent autophagy indicator system was purchased from Han heng Biotechnology Co., Ltd (Shanghai, China). LC3 specific monoclonal antibodies were purchased from Abcam(Cambridge, MA, United States).

### Cell culture and experimental groups

The cells were cultured in RPMI-1640 medium (PAA Laboratories GmbH, Austria) supplemented with 10% fetal bovine serum (FBS) in a humidified atmosphere containing 5% CO_2_ at 37°C. Cells were treated with radiation alone, SSd alone, or a combination of radiation and SSd, respectively. Radiation was performed at a dose of 2Gy (6 MV, and a dose rate of 400 cGy/min) by using an X-ray linear accelerator at room temperature. SSd was also administered at concentrations (3µg/ml) as described previously 13. SSd was added to the cultures at 2 h before radiation. Chloroquine (CQ) (25 μmol/L) or mTOR inhibitor (MHY1485, 20nmol/L) was added 4 hours prior to radiation or ssd intervention. Control cultures received a carrier solvent consisting of 0.1% DMSO.

### Cell viability determination

The experimental steps for detecting cell activity refer to our previous methods 14. Cells were seeded in a 96-well plate (1×10^4^ cells/well) and incubated at 37°C in 5% CO_2_ for different periods as desired. MTT solution was added and the cells were incubated for another 4 h. Supernatants were removed and formazan crystals were dissolved in 200 µl of DMSO. Optical density was determined at 490 nm by using a multi-microplate test system (POLARstar OPTIMA, BMG Labtechnologies, Germany). Relative cell viability: (average OD value of the intervention group / average OD value of the control group) × 100%.

### Cell survival analysis

The cells of each experimental group were treated in a 6-well plate, digested into single cell suspension by trypsinization, and 6-well plates were inoculated at a density of 200 cells per well, placed in a cell culture incubator for 10 days. The culture solution was immersed twice in PBS, fixed in 4% paraformaldehyde for 15 min, and immersed twice in PBS. The cells were stained with crystal violet for 20 min, washed slowly with tap water until they did not fade. After drying in a ventilated place, photographs were taken and the number of clones was counted,and the colonies with more than 50 cells were counted. The clonal formation rate = number of colonies in the intervention group / number of control colonies × 100%.

### Transmission electron microscope

The autophagy in hepatocarcinoma cells of each experimental group was observed under transmission electron microscope. The cells of each experimental group were collected, digested with 2.5g/L trypsin, centrifuged at 3000 r/min, washed with PBS, collected in EP tubes, then fixed with 25 g/L glutaraldehyde, with 10 g/L citric acid, and dehydrated by graded ethanol, infiltrated and embedded in epoxy resin. After slicing by the ultra microtome, cells were stained by uranyl acetate and lead citrate, and observed under a transmission electron microscope.

### Western blot analysis

Refer to our previous experimental methods 13. Add about 100μl of RIPA lysate containing protease and phosphatase inhibitor per 1×10^6^ cells, lyse on ice for 30 min. After the lysis, the cell lysate was placed in a centrifuge at 12000 g at 4°C, centrifuged for 30 min, and the supernatant was collected and quantified by BCA method. Finally, add 5×loading buffer, boil water for 10 min and then electrophoresis. The blots were probed with antibodies against LC3 (diluted 1:2000). Blot images were obtained from the FX5 spectral imaging system and the optical density of the bands was measured by Image J software (NIH, Bethesda, MD, USA).The membranes were re-probed for β-actin as loading control.

### mRFP-GFP-LC3 fluorescence method for tracing autophagy

The 7721 cells were transfected with mRFP-GFP-LC3 plasmid according to the manufacturer's instructions. 24 h later, different conditions were intervened and autophagy was observed under laser confocal microscopy. The number of puncta in liver cancer cells was counted to evaluate the level of autophagy.

### Statistical analysis

Quantitative data were presented as the mean ± standard error of the mean (SEM) and analyzed by one-way ANOVA. Statistical analyses were performed using SPSS software (version 17.0). Tukey's post hoc analyses were conducted to assess the difference between groups. Data were considered significant if *P* < 0.05.

## Results

### Effects of SSd and radiation on hepatoma cell growth

**Figure 1:**
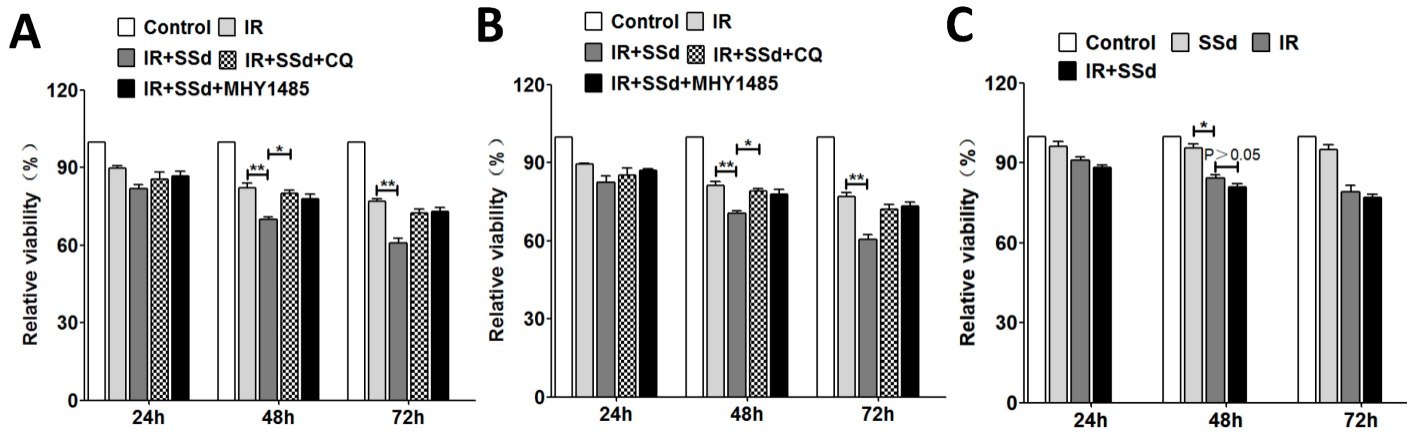
** Changes in cell viability after different interventions.** A. MTT demonstrate that the effect of different interventions on the viability of SMMC-7721 cells; B. The effect of different interventions on the viability of MHCC97L cells; C. The effect of different interventions on the viability of normal hepatocytes. (*P< 0.05,**P< 0.01).

As our previously described that the cell viabilities of SMMC-7721 cells were significantly reduced in a dose-dependent manner and time-dependent manner after treated with SSd, radiation, or a combination of SSd and radiation^13^. In this study, we further selected SSd (3μg/mL) and radiation (2 Gy) to act on SMMC-7721 or MHCC97L cells to study its effect on cell activity and autophagy levels. MTT demonstrated that radiation could inhibit the growth of SMMC-7721 cells and MHCC97L cells obviously, and in the combined group, SSd can further enhance the inhibitory effect of radiation on liver cancer cells. As shown in Figure 1. At different time points, differences of relative cell viability between the combined group and radiation group were statistically significant. After the addition of CQ or MHY1485, the radiosensitization effect of SSd on SMMC-7721 cells was significantly reduced. Relative cell viability increased from 70% to 80% or 77.8% in the combined group at 48 h after treatment. (Figure 1 A, *P*< 0.05). Similarly, the reduction in radiosensitization of SSd can also be observed in MHCC97L cells after the addition of CQ or MHY1485 (Figure 1 B, *P*< 0.05). In addition, we further studied the effect of SSd on the radiosensitivity of normal hepatocytes. The relative cell viability of normal hepatocytes was significantly lower than that of the control group after treated with 2 Gy radiation. Although 3μg/mL of SSd also had a certain inhibitory effect on normal hepatocytes, there was no significant difference compared with the control group, and this concentration of SSd has no significant enhancement in radiosensitivity to normal hepatocytes (Figure 1 C, *P*>0.05).

### Effect of SSd and radiation on cell colony formation

**Figure 2:**
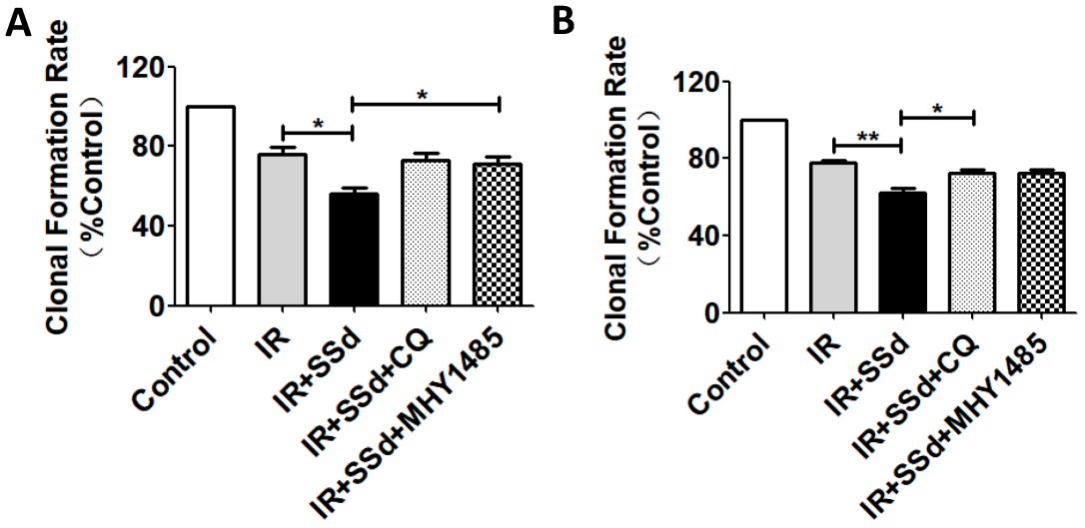
** Changes in clonal formation rate after different interventions.** A more pronounced decrease of colony formation rate was observed in the combined treatment group than that of in radiation groups. Meanwhile, colony formation rate of combined treatment group was increased after the intervention of CQ or MHY1485. A. Colony formation rate changes of SMMC-7721 cells; B. Colony formation rate changes of MHCC97L cells (*P< 0.05, **P< 0.01).

When cells were exposed to 2 Gy radiation alone, The clonal formation rate of SMMC-7721 cells was 76.1%. However, when SSd (3 μmol/L) was added, a more pronounced decrease of cell survival was observed in the combined treatment group than that of in radiation groups (Figure 2A, *P<*0.05). Meanwhile, it was found that the clonal formation rate of combined treatment group was increased from 56.4% to 72.8% after the intervention of CQ, and it was 71.2% after the intervention of MHY1485 (Figure 2A, *P<*0.05). In addition, similar cell clone formation interventions can also be observed in MHCC97L cells (Figure 2B). These results provided additional evidence that radiotherapy sensitization of SSd in SMMC-7721 cells or MHCC97L cells can be partially reversed by CQ or MHY1485.

### Effects of SSd and radiation on the formation of autophagy under transmission electron microscope

**Figure 3:**
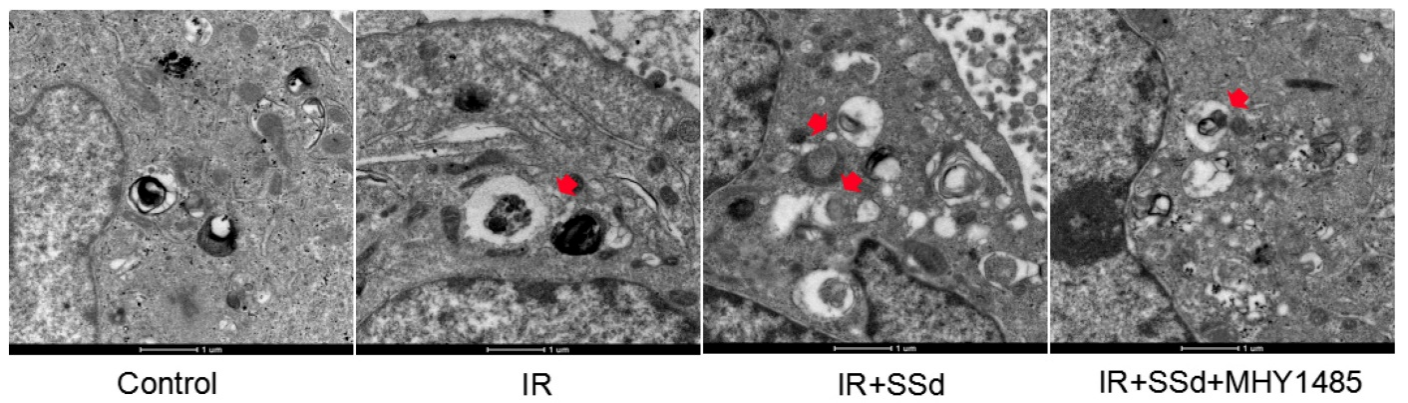
** Formation of autophagy in Hepatoma Cells was observed under transmission electron microscope.** Autophagosomes of SMMC-7721 cells in the combined treatment group increased in large quantities, as indicated by the red arrow. However, almost no autophagosomes appeared in MHY1485 groups (×16500).

This study further tested the influence of the SSd intervention on the autophagosome formation in hepatoma cells under transmission electron microscope. Results indicated that after SSd processing, autophagosomes of SMMC-7721 cells in the combined treatment group increased in large quantities, as indicated by the red arrow. However, almost no autophagosome appeared in the control or MHY1485 groups. The above results indicated that SSd increases radiation-induced autophagosome formation in hepatoma cells as seen in Figure 3.

### Effect of SSd and radiation on LC3-II expression

**Figure 4:**
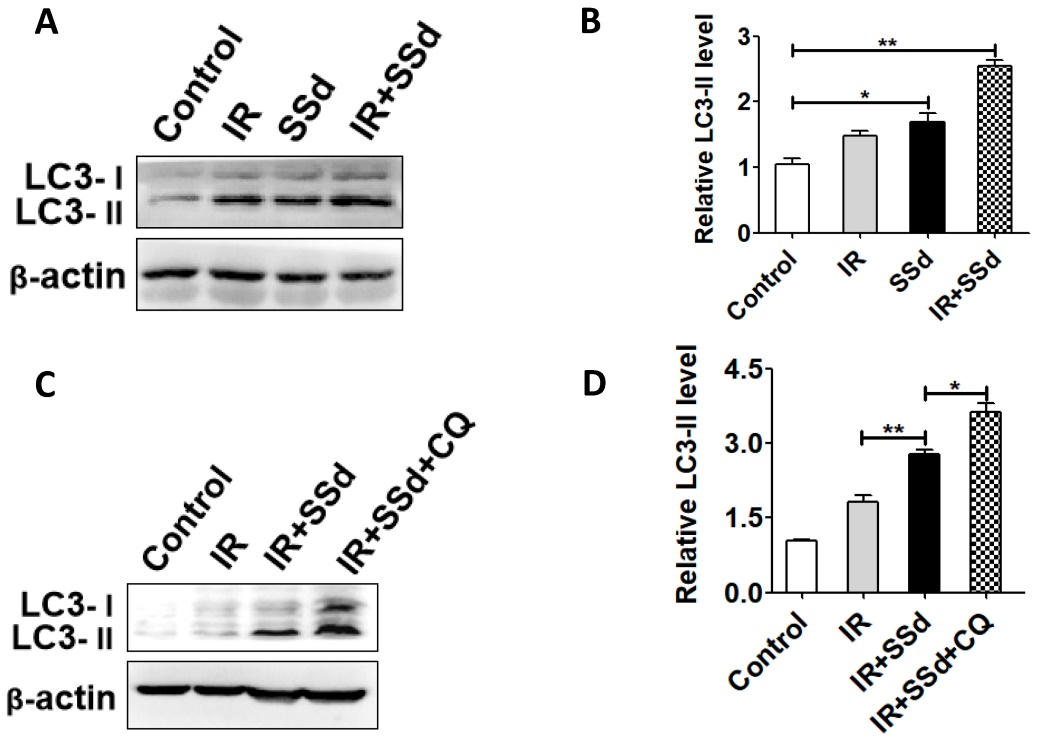
** LC3-II expressions were detected by western blot.** A. Western blot analysis of the LC3-II levels in SMMC-7721 cells after treated with different interventions; B. Relative LC3-II expression in SMMC-7721 cells; C. Western blot analysis of the LC3-II levels in SMMC-7721 cells after addition of CQ; D. Relative LC3-II expression in SMMC-7721 cells after addition of CQ. (*P< 0.05, **P< 0.01).

As shown in Figure 4, the western blot analysis presented that radiation significantly increased LC3-II levels. Especially after the addition of SSd, LC3-II levels is further increased (*P<*0.05). This indicates that SSd can increase radiation-induced autophagy of SMMC-7721 hepatoma cells. In order to further clarify that the up-regulation of LC3-II protein is due to the induction of autophagy by SSd rather than the inhibition of autophagosome degradation, the trial uses a lysosomal inhibitor CQ to co-treat SMMC-7721 hepatoma cells with SSd. The results showed that the LC3-II level was further increased after adding CQ compared with the SSd group, indicating that the upregulation of LC3-II protein after treatment with SSd was due to its induction of autophagy rather than inhibiting the degradation of autophagosomes.

### Effects of SSd and radiation on the formation of autophagy under laser confocal microscopy

**Figure 5:**
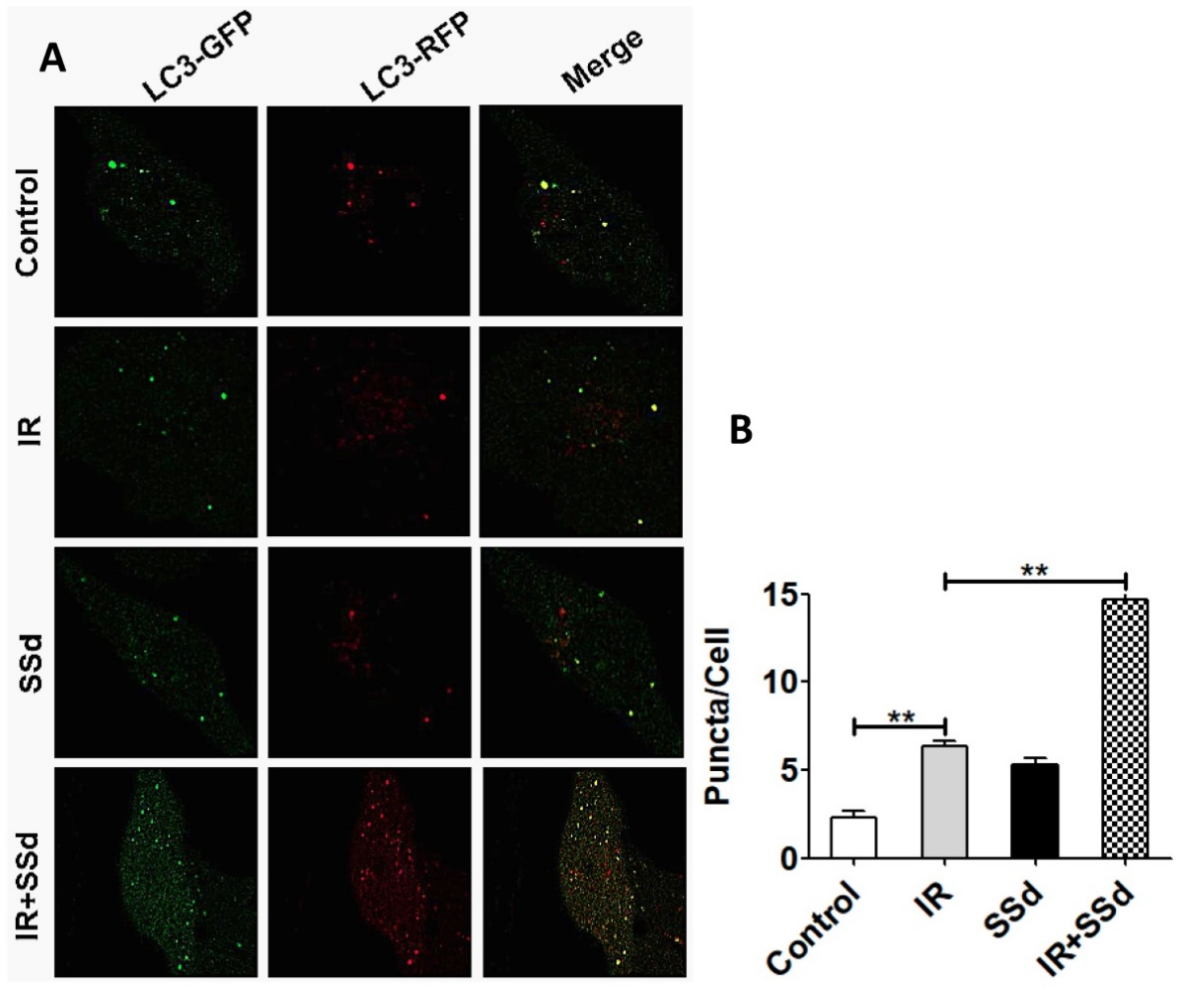
** Changes of the number of autophagosomes in SMMC-7721 cells after different interventions.** The number of autophagosomes was 2.33±0.33 in the control group. After the addition of SSd, the number of autophagosomes in the combined group is higher than that of radiation alone group. Representative photographs obtained from each group (A) and statistical analysis from all cells in each group (B). (**P< 0.01).

In order to further quantify the effect of the combination of SSd and radiation on the formation of autophagy in SMMC-7721 cells, this study further tested autophagosome formation by the mRFP-GFP-LC3 fluorescence method and the number of puncta in the cells reflects the activity of autophagy. Results indicated that the number of autophagosomes was 2.33±0.33 in the control group, 6.33±0.33 in the radiotherapy group, and there was a significant difference between the two groups (Figure 5, *P*<0.01). After the addition of SSd, the number of autophagosomes is as high as 14.67 ± 0.88, which is significantly higher than that of the radiation alone group (*P*< 0.01). The results show that SSd can significantly increase the autophagic formation of SMMC-7721 hepatoma cells induced by radiation (Figure 5).

## Discussion

Autophagy is a highly conserved evolutionary process that is ubiquitous in cells and is involved in the regulation of many important physiological and pathological processes. Studies have shown that autophagy has a dual role in tumor formation: in the early stage of tumors, autophagy inhibits the growth of tumor cells, cells clear the damaged organelles through autophagy, reduce inflammation, maintain cell homeostasis, and protect normal cell growth. In the advanced stage of tumors, autophagy is an important cause of radiation resistance and drug resistance in tumor cells and effective regulation of cellular autophagy can enhance the sensitivity of radiotherapy 16,17.

Recent studies have shown that saikosaponin-d has many pharmacological activities such as anti-tumor, induced autophagy, radiosensitization and immune regulation 13,14. However, the effect of SSd on the level of autophagy in liver cancer cell radiotherapy has not reported. In this study, MTT and cell colony formation demonstrate that radiation could inhibit the growth of SMMC-7721 cells and MHCC97L cells obviously, and in the combination group, SSd can further enhance the inhibitory effect of radiation on liver cancer cells. Furthermore, under transmission electron microscope, the autophagy of SMMC-7721 in the combined group was significantly increased compared with the control or the radiation group. After the addition of the autophagy inhibitor chloroquine or MHY1485, the induced autophagy and radiosensitization effect of SSd on liver cancer cells were significantly reduced.

In addition, we analyzed the expression of LC3 protein by western blot. There are two forms of LC3 protein in cells: LC3-I (18KD) and LC3-II (16KD). When autophagy does not occur in the cells, the C-terminus of the intracellular LC3 protein is cleaved by the Atg4 protease to become a soluble LC3-I protein, which is scattered in the cytoplasm. When autophagy occurs in the cells, the LC3-I protein undergoes ubiquitin-like processing and is coupled with phosphatidylethanolamine (PE) on the surface of the autophagosome membrane to form LC3-II protein. The LC3-II protein binds and is stably localized on the autophagosome membrane, so it is used as a marker for autophagosomes. The content of LC3-II protein is positively correlated with the number of autophagosomes, which can reflect the number of autophagosomes to some extent. Therefore, the expression intensity of LC3-II protein can be used to judge Activity of autophagy 18,19. In this experiment, the changes of LC3-II protein were tested after treated with the combination of SSd and radiation. The results showed that the effect of SSd significantly increased the expression of radiation-induced LC3-II. In order to further clarify that the up-regulation of LC3-II protein is due to the induction of autophagy by SSd rather than the inhibition of autophagosome degradation, the trial uses a lysosomal inhibitor CQ to co-treat SMMC-7721 liver cancer cell with SSd. The results showed that the LC3-II level was further increased after adding CQ compared with the SSd group, indicating that the upregulation of LC3-II protein after treatment with SSd was due to its induction of autophagy rather than inhibiting the degradation of autophagosomes. Combined with microscopic observation and Western- blot analysis, it was confirmed that SSd increased the radiosensitivity of hepatoma cells by inducing autophagy.

Furthermore, in order to further quantitatively observe the autophagosome formation in hepatoma cells under different intervention conditions, we observed the changes in the number of autophagosomes under laser confocal microscopy. The results showed that the number of autophagosomes in SMMC-7721 hepatoma cells was significantly higher than that in the control group after radiotherapy, and the number of autophagosomes was further increased with the addition of SSd, which was significantly different from the radiotherapy group alone.The results show that SSd can significantly increase the autophagic formation of SMMC-7721 cells induced by radiation.

The mammalian target of rapamycin (mTOR) is an atypical serine/threonine protein kinase, which is the receptor for amino acids, ATP and hormones in cells. mTOR affects autophagy and has a gating effect in autophagy by causing changes in autophagy-related gene (ATG). Many oncogenes and tumor suppressor genes regulate cellular autophagy by acting on mTOR^20^,^21^.This experiment indicates that SSd can promote the inhibition of radiation on hepatoma cell proliferation and clonal formation. However, this effect of SSd on radiation was reversed after the addition of the autophagy inhibitor chloroquine or mTOR agonist. These data indicate that inhibition of autophagy can reduce the radiosensitizing effect of SSd on hepatoma cells.

However, the current results are far from enough to understand the specific mechanism of SSd in the radiosensitization of liver cancer. Although many studies have shown that SSd can induce tumor cell apoptosis 22,23 and autophagic death mostly occurs in the absence of apoptosis 24. Therefore, the inhibitory effect of SSd on tumors and whether there is an interaction between apoptosis and autophagy requires further research to confirmation. The occurrence and development of liver cancer is a complex process involving multiple signaling pathways. In-depth study of the sensitization effect of SSd on liver cancer radiotherapy may provide new ideas for drug therapy of liver cancer.

## References

[1] Kim DY, Han KH (2012). How to improve treatment outcomes for hepatocellular carcinoma of intermediate andadvanced stage. Dig Dis.

[2] Cong WM, Bu H, Chen J, Guideline Committee (2016). Practice guidelines for the pathological diagnosis of primary liver cancer: 2015 update. World J Gastroenterol.

[3] Jemal A, Bray F, Center MM (2011). Global cancer statistics. CA Cancer J Clin.

[4] Bruix J, Gores GJ, Mazzaferro V (2014). Hepatocellular carcinoma: clinical frontiers and perspectives. Gut.

[5] Sapisochin G, Barry A, Doherty M (2017). Stereotactic body radiotherapy vs. TACE or RFA as a bridge to transplant in patients with hepatocellular carcinoma. An intention-to-treat analysis. J Hepatol.

[6] Su TS, Liang P, Lu HZ (2016). Stereotactic body radiation therapy for small primary or recurrent hepatocellular carcinoma in 132 Chinese patients. J Surg Oncol.

[7] Nam HY, Han MW, Chang HW (2013). Prolonged autophagy by MTOR inhibitor leads radioresistant cancer cells into senescence. Autophagy.

[8] Gewirtz DA (2014). The autophagic response to radiation: relevance for radiation sensitization in cancer therapy. Radiat Res.

[9] Galluzzi L, Pietrocola F, Bravo-San Pedro JM (2015). Autophagy in malignant transformation and cancer progression. EMBO J.

[10] Fuchs Y, Steller H (2015). Live to die another way: modes of programmed cell death and the signals emanating from dying cells. Nat Rev Mol Cell Biol.

[11] Kuwahara Y, Oikawa T, Ochiai Y (2011). Enhancement of autophagy is a potential modality for tumors refractory to radiotherapy. Cell Death Dis.

[12] Chang SH, Minai-Tehrani A, Shin JY (2012). Beclin1-induced autophagy abrogates radioresistance of lung cancer cells by suppressing osteopontin. J Radiat Res.

[13] Wang BF, Wang XJ, Kang HF (2014). Saikosaponin-D enhances radiosensitivity of hepatoma cells under hypoxic conditions by inhibiting hypoxia-inducible factor-1α. Cell Physiol Biochem.

[14] Wang BF, Dai ZJ, Wang XJ (2013). Saikosaponin-d increases the radiosensitivity of smmc-7721 hepatocellular carcinoma cells by adjusting the g0/g1 and g2/m checkpoints of the cell cycle. BMC Complement Altern Med.

[15] Wong VK, Li T, Law BY (2013). Saikosaponin-d, a novel SERCA inhibitor, induces autophagic cell death in apoptosis-defective cells. Cell Death and Disease.

[16] Gewirtz DA (2014). An autophagic switch in the response of tumor cells to radiation and chemotherapy. Biochem Pharmacol.

[17] Nagelkerke A, Bussink J, Geurts-Moespot A (2015). Therapeutic targeting of autophagy in cancer. Part II: pharmacological modulation of treatment-induced autophagy. Semin Cancer Biol.

[18] Noboru Mizushima, Tamotsu Yoshimori, Beth Levine (2010). Methods in Mammalian Autophagy Research. Cell.

[19] Li R, Ma M, Li L (2018). The Protective Effect of Autophagy on DNA Damage in Mouse Spermatocyte-Derived Cells Exposed to 1800 MHz Radiofrequency Electromagnetic Fields. Cell Physiol Biochem.

[20] Chen L, Jiang Z, Ma H (2016). Volatile Oil of Acori Graminei Rhizoma-Induced Apoptosis and Autophagy are dependent on p53 Status in Human Glioma Cells. Sci Rep.

[21] Jing K, Song KS, Shin S (2011). Docosahexaenoic acid induces autophagy through p53/AMPK/mTOR signaling and promotes apoptosis in human cancer cells harboring wild-type p53. Autophagy.

[22] Shi W, Xu D, Gu J (2018). Saikosaponin-d inhibits proliferation by up-regulating autophagy via the CaMKKβ-AMPK-mTOR pathway in ADPKD cells.

[23] Li Y, Cai T, Zhang W (2017). Effects of Saikosaponin D on apoptosis in human U87 glioblastoma cells. Mol Med Rep.

[24] Ding ZB, Shi YH, Zhou J (2008). Association of autophagy defect with a malignant phenotype and poor prognosis of hepatocellular carcinoma. Cancer Res.

